# Picture of the Favourable Immune Profile Induced by Anti-SARS-CoV-2 Vaccination

**DOI:** 10.3390/biomedicines9081035

**Published:** 2021-08-18

**Authors:** Paola Lanuti, Claudia Rossi, Ilaria Cicalini, Laura Pierdomenico, Verena Damiani, Daniela Semeraro, Sara Verrocchio, Piero Del Boccio, Adelia Evangelista, Annalina Sarra, Mirco Zucchelli, Giuseppina Bologna, Pasquale Simeone, Giulia Catitti, Federica Di Marco, Simone Stefanetti, Simone Vespa, Bruna Sinjari, Ines Bucci, Vincenzo De Laurenzi, Tonio Di Battista, Liborio Stuppia, Damiana Pieragostino

**Affiliations:** 1 Center for Advanced Studies and Technology (CAST), G. d’Annunzio University of Chieti-Pescara, 66100 Chieti, Italy; paola.lanuti@unich.it (P.L.); claudia.rossi@unich.it (C.R.); ilaria.cicalini@unich.it (I.C.); laura.pierdomenico@unich.it (L.P.); verena.damiani@unich.it (V.D.); d.semeraro@unich.it (D.S.); sara.verrocchio@gmail.com (S.V.); p.delboccio@unich.it (P.D.B.); m.zucchelli@unich.it (M.Z.); giuseppina.bologna@hotmail.it (G.B.); simeone.pasquale@gmail.com (P.S.); catittig@gmail.com (G.C.); dmfederica95@gmail.com (F.D.M.); simo.stefanetti94@hotmail.it (S.S.); sv85@libero.it (S.V.); ines.bucci@unich.it (I.B.); delaurenzi@unich.it (V.D.L.); stuppia@unich.it (L.S.); 2 Department of Medicine and Aging Science, G. d’Annunzio University of Chieti-Pescara, 66100 Chieti, Italy; 3 Department of Psychological, Health and Territory Sciences, School of Medicine and Health Sciences, G. d’Annunzio University of Chieti-Pescara, 66100 Chieti, Italy; 4 Department of Innovative Technologies in Medicine and Dentistry, G. d’Annunzio University of Chieti-Pescara, 66100 Chieti, Italy; b.sinjari@unich.it; 5 Department of Pharmacy, G. d’Annunzio University of Chieti-Pescara, 66100 Chieti, Italy; 6 Department of Philosophical, Pedagogical and Economic-Quantitative Sciences, G. d’Annunzio University of Chieti-Pescara, 65127 Pescara, Italy; adelia.evangelista@unich.it (A.E.); asarra@unich.it (A.S.); tonio.dibattista@unich.it (T.D.B.)

**Keywords:** SARS-CoV-2, vaccines, anti-S1 IgG, spike-specific T-cells

## Abstract

COVID-19 pandemic has hit people’s health, economy, and society worldwide. Great confidence in returning to normality has been placed in the vaccination campaign. The knowledge of individual immune profiles and the time required to achieve immunological protection is crucial to choose the best vaccination strategy. We compared anti-S1 antibody levels produced over time by BNT162b2 and AZD1222 vaccines and evaluated the induction of antigen-specific T-cells. A total of 2569 anti-SARS-CoV-2 IgG determination on dried blood spot samples were carried out, firstly in a cohort of 1181 individuals at random time-points, and subsequently, in an independent cohort of 88 vaccinated subjects, up to the seventeenth week from the first dose administration. Spike-specific T-cells were analysed in seronegative subjects between the two doses. AZD1222 induced lower anti-S1 IgG levels as compared to BNT162b2. Moreover, 40% of AZD1222 vaccinated subjects and 3% of BNT162b2 individuals resulted in seronegative during all the time-points, between the two doses. All these subjects developed antigen-specific T cells, already after the first dose. These results suggest that this test represents an excellent tool for a wide sero-surveillance. Both vaccines induce a favourable immune profile guaranteeing efficacy against severe adverse effects of SARS-CoV-2 infection, already after the first dose administration.

## 1. Introduction

A massive vaccination campaign against SARS-CoV-2 is being carried out throughout the world with different vaccines, thus making of great importance the acquisition of as much information as possible on the ability and time required for these compounds to generate an immune response in order to choose the best vaccination strategy. In fact, while in the UK the selected strategy has been to delay the second dose, allowing a wider administration of the first dose, the majority of other European countries followed the two-doses strategy, according to the schedule suggested by clinical trials. At present, however, many countries are considering or have already implemented a delay in the administration of the second dose.

In this study, we compared the efficiency of the Pfizer–BioNTech COVID-19 (BNT162b2) vaccine and the ChAdOx1 nCoV-19 vaccine (AZD1222) by AstraZeneca in the production of anti-S1 IgG and analysed the immune profiling of IgG seronegative vaccinated subjects by the study of SARS-CoV-2 specific T-cells. Briefly summarizing their characteristics, the Pfizer–BioNTech COVID-19 vaccine is an mRNA vaccine encoding a P2 mutant spike protein (PS 2), where two proline substitutions (2P) at the apex of the central helix and HR1 allow to retain the S proteins in the antigenically optimal pre-fusion conformation. In particular, the BNT162b2 vaccine is formulated as an RNA–lipid nanoparticle of nucleoside-modified mRNA (modRNA) [1]. The ChAdOx1 nCoV-19 vaccine (AZD1222), developed at Oxford University, consists of a replication-deficient chimpanzee adenoviral vector (ChAdOx1), containing the SARS-CoV-2 structural surface glycoprotein antigen (spike protein; nCoV-19) gene [2,3]. After the implementation of vaccination programs, serological surveys of large representative populations for immunoglobulin G (IgG) antibodies to SARS-CoV-2 is becoming an important tool to evaluate the expression and the duration of antibody responses as well as the percentage of responders [4,5]. Many studies are appearing in the literature on the effects of vaccines. It was recently demonstrated that the titer of neutralizing antibodies was markedly higher in response to the BNT162b2 vaccine than after natural infection [6]. Most current serological tests require serum or plasma samples collected by venepuncture, which is laborious and requires trained healthcare staff, thus being less suitable for large-scale monitoring of the immune response [4]. In this context, capillary blood collected as dried blood spot (DBS) samples represents a valid alternative to plasma/serum collection for IgG detection [7], especially in a large-scale population screening. The level of antibodies detected in such biosamples is widely correlated to the level of antibodies detected in serum/plasma as already demonstrated by Zava et al. [8].

The generation of neutralizing antibodies cooperates with B- and T-cell responses in the adaptive immune responses directed against the spike glycoprotein (S) [9], inducing long-term protection from severe respiratory infection (>6–17 years) [10]. This long-lasting antiviral immunity requires the enrolment of T-cells, both CD4+ and CD8+, and the generation of effective T cell memory that can also serve as a sensitive biomarker of previous exposures to the spike glycoprotein [10]. We, therefore, also evaluated T-cell responses in subjects seronegative after vaccine administration.

## 2. Materials and Methods

### 2.1. Patients

Samples were collected from the Center for Advanced Studies and Technology (CAST), “G. d’Annunzio” University of Chieti-Pescara, where is active the “Newborn screening laboratory” for the collection and management of the BDS samples. Informed consent was collected for all patients enrolled in the study (following point 32 of the Declaration of Helsinki 2013). Venous withdrawals were collected following the Ethical Committee approval N. 16 of 1 July 2021.

One thousand two hundred and sixty-nine subjects were enrolled, as reported in Table S1. Among these donors, 1181 were evaluated at a single time point, and were divided into four groups (2–70 years old); more specifically 398 individuals received one dose and 149 received two doses of AZD1222 vaccine, 257 received two doses of BNT162b2 vaccine, 235 subjects had a resolved natural SARS-CoV-2 infection, and 147 donors were not vaccinated and did not report previous infection (control group).

Moreover, an independent casuistry of 88 donors was weekly monitored until the seventeenth week. These 88 volunteers underwent a time course study and IgG levels were evaluated before and after the administration of the vaccines. In detail, 36 participants (75% female, mean age 35.1 ± 9.4 [range 23 to 61]), who received the first and second BNT162b2 vaccine dose, were monitored; among them, only one donor was seropositive for COVID-19. Furthermore, 52 participants (59.6% female, mean age 40.8 ± 9.2 [range 25 to 59]) receiving the first and the second AZD1222 vaccine dose were also observed; among them, three donors were seropositive for COVID-19. Seropositive patients were included in the bean plot graph, but they were excluded from statistical analyses. More specifically, the AZD1222 participants were re-called weekly for a total of 17 weeks, while BNT162b2 participants were weekly recalled until 35 days after second dose administration and every 14 days until the seventeenth week after the first injection of vaccine for re-determination of IgG levels. For the flow cytometry activated T cell analysis, 15 control subjects (8 males, 53.33%, and 7 females, 46.67%, 45.93 ± 11.84 [range 24–69]), and 23 donors who had received the first dose of AZD1222 vaccine (11 males, 47.83%, and 12 females, 52.17%, mean age 44.04 ± 7.68 [range 29–57]), one donor after receiving the first dose of BNT162b2 vaccine and 15 fully AZD1222 vaccinated donors (7 males, 46.67%, and 8 females, 53.33%, mean age 42.93 ± 8.48 [range 29–56]) were enrolled. No concomitant pathologies were declared by the enrolled donors.

### 2.2. Anti-S1 Spike IgG Measurement

IgG antibodies to SARS-CoV-2 were measured by a fully automated solid phase DELFIA (time-resolved fluorescence) immunoassay in a few drops of blood collected by finger-prick and dried on filter paper, by using GSP^®^/DELFIA^®^ Anti-SARS-CoV-2 IgG kit time-resolved fluoroimmunoassayon a GSP instrument (PerkinElmer). IgG levels are calculated as a ratio of fluorescence of the sample over the calibrator. The over-described sample procedure is minimally invasive and thus more acceptable to subjects than the venous puncture, usually applied to measure IgG antibodies to SARS-CoV-2 from serum or plasma. Therefore, the DBS specimen represents the ideal sample for population-based screening methods and an interesting tool for the constant monitoring of IgG antibodies to SARS-CoV-2 in the vaccinated population. The test was screened as positive subjects having IgG levels above the laboratory 1.2 cut-off.

### 2.3. Functional Data Analysis, FDA and Statistics

We analysed IgG levels as Functional Data Analysis (FDA) for each unit representing the individuals treated with a specific vaccine. We also consider that the units represent a random sample of the observed curves. Details of the FDA approach are fully reported in Supplementary Materials.

All statistical tests were performed using GraphPad Prism 9 (GraphPad Software, San Diego, CA, USA), XLSTAT2021 (Addinsoft, New York, NY, USA), and MedCalc V. 20 (MedCalc Software Ltd., Ostend, Belgium). Details are reported in Supplementary Materials.

### 2.4. PBMC Isolation, Stimulation and Staining for Flow Cytometry Analysis

Peripheral blood mononuclear cells (PBMC) were stimulated with a pool of spike peptides (PepTivator S, cat. 130-126-701, PepTivator S1, cat. 130-127-048, Peptivator S+, cat. 130-127-312, MiltenyiBiotec, Bergisch Gladbach, Germany) at the recommended concentrations for 16 h (37 °C, 5% of CO_2_), while negative controls were treated with the same amount of vehicle [9,11,12]. After 2 h of stimulation, samples were treated with 6.5 µL GolgiStop (554724, BD Biosciences, La Jolla, CA, USA). The TCR-dependent activation-induced marker (AIM) assay [13] and flow cytometry with intracellular cytokine staining assays (ICS) were carried out as reported [12,13]. A representative example of a gating strategy is depicted in Figure S1. Reagent list for Flow cytometry analysis are reported in Table S2.

## 3. Results

### 3.1. Evaluation of Anti-SARS-CoV-2 IgG Levels at Random Time Points

**Figure 1:**
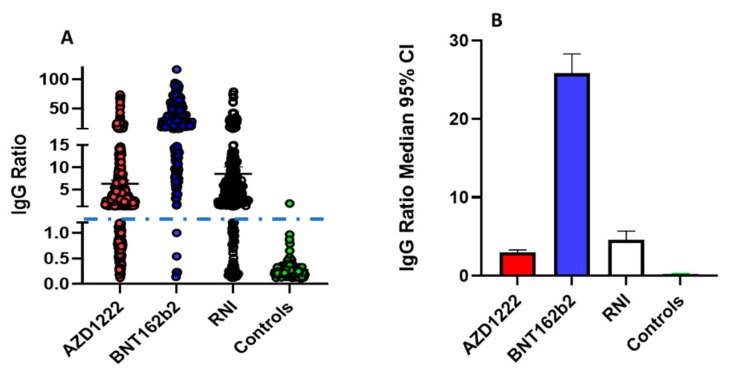
Panel (**A**) dot plot of the four groups analysed: Red dots IgG ratio for AZD1222 vaccinated donors, blue dots for BNT162b2 vaccinated donors, white RNI donors, and green dots controls subjects. Blue dashed line shows the positivity cut-off ratio. Panel (**B**) shows the median and 95% CI of IgG ratio for the groups in the study with the same colour code of panel (**A**).

Initially, a survey analysis of anti-SARS-CoV-2 IgG levels was carried out on 1269 individuals. As shown in Figure 1A (and detailed depending on the time in Figure S2), IgG levels cluster in three distinct groups: negative (0–1.2), low positives (1.2–15), and high positives (15–100). As shown in Figure S2 after the second dose high percentage of AZD1222 vaccinated patients become positive; moreover, a downward trend is observed after the thirtieth day fromBNT162b2 vaccination. Figure 1B clearly shows the median IgG ratio with a confidence interval of 95%, confirming the same trend highlighted in panel A. As expected, the majority of subjects who received two doses of BNT162b2 are in the high positive group while the majority of RNIs and subjects receiving AZD1222 are in the low positive group, suggesting that the vaccine potentially yields antibody levels comparable to previously infected subjects. However, these data are biased by collection at random time points from infection or vaccination and by the fact that all subjects had received two doses of BNT162b2. We, therefore, performed a time-course analysis of IgG production on a different cohort of subjects.

### 3.2. Anti-SARS-CoV-2 IgG Functional Grow Curves before the Second Dose Administration

**Figure 2:**
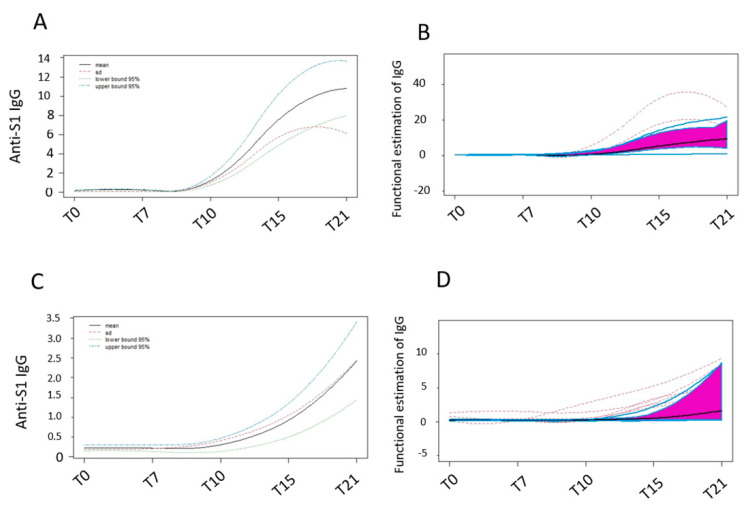
Panel (**A**,**C**) show the mean level after the first dose administration (black line) of vaccines and the standard deviation between the IgG level (represented by the dashed red line) in BNT162b2 and AZD1222 vaccine, respectively. Panel (**B**,**D**) show the functional boxplot for BNT162b2 and AZD1222 IgG levels, respectively. The purple areas highlight the amplitude of 50% of the most central observation while the two outer blue curves correspond to the maximum and minimum curve of the dataset.

IgG production was analysed before and every seven days after the administration of the first dose of either BNT162b2 or AZD1222 vaccines, thus up to the twenty-first day when the BNT162b2 group received the second dose. We analysed the IgG ratio using functional grow curves that allow the transformation of discrete data, observed repeatedly over time, into continuous data, through a mathematical function. Figure 2 panel A shows that the mean level increases after the first dose administration (black line) of the BNT162b2 vaccine, highlighting a constant growth, especially between T10 and T15, with a slight slowdown between T15 and T21, which is well placed with the boost planned on the twenty-first day. Interestingly, the standard deviation between the IgG levels (represented by the dashed red line) tends to drop after 21 days suggesting that levels measured in different subjects tend to converge resulting in a more homogenous population.

In Figure 2B, the functional boxplot for BNT162b2 IgG levels is reported, showing the amplitude of 50% of the most central observations into the purple area. Moreover, the two outer blue curves (i.e., the highest and the lowest) correspond to the maximum and minimum curve of the dataset and show high variability in the central time points. This graph also shows how the two outliers (red dashed lines) tend to conform to others on the twenty-first day. Again, the AZD1222 vaccine shows a different behaviour as highlighted in Figure 2C, since as the levels keep rising the standard deviation (dashed red line and black line) also increases, thus indicating that the population is becoming more heterogeneous and that the antibody levels of responders increasingly differ from that of non-responders. This evidence is reinforced by results reported in the functional boxplot in panel D, showing a significantly higher interquartile difference (purple area) and suggesting a greater variability and low IgG levels, as indicated by the median approaching the first quartile. It is also noted that there are cases that have not yet responded to the vaccine, as shown by the blue line that drops below zero.

### 3.3. Time Course Monitoring of Anti-SARS-CoV-2 IgG Levels

**Figure 3:**
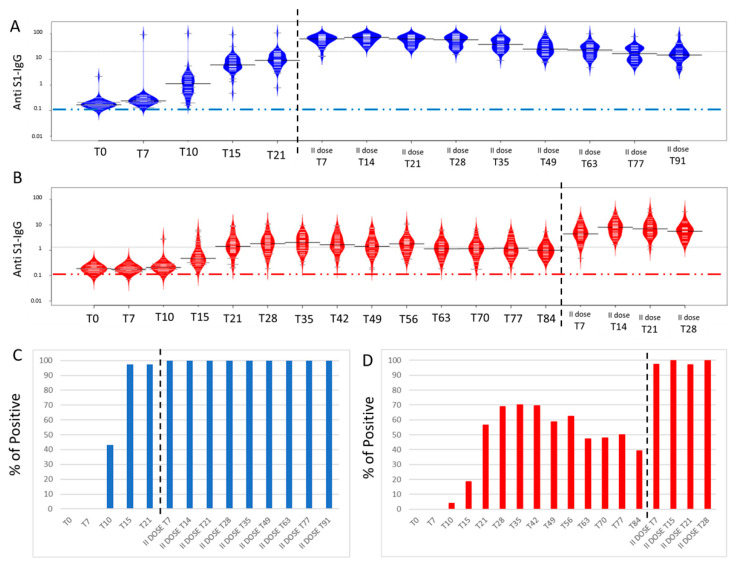
Panel (**A**): Beanplot of anti-S1 IgG levels after BNT162b2 vaccine administration. Dashed grey line is the median of analyses, while the dashed blue line indicates the cut-off of positivity. Panel (**B**): Beanplot of anti-S1 IgG levels after AZD1222 vaccine administration. Dashed grey line shows the median of analyses, while dashed red line indicates the cut-off of positivity. Panel (**C**,**D**): Frequency of positives patients in percentages. Blue bars represent the BNT162b2 vaccine (Panel (**C**)), while Red bars show the AZD1222 vaccine (Panel (**D**)). Dashed black vertical line indicates the time of the second dose vaccine administration in all panels.

The BNT162b2 and AZD1222 vaccinated participants were further monitored up to the seventeenth week. As shown in Figure 3A, for the BNT162b2 vaccine, IgG levels start to rise above the cut off level after 10 days from the first injection and by day 15 the majority of subjects shows IgG levels above the threshold reaching an average of 7.76 ± 5.6, the significance of Dunn’s test, as reported in Table S3. AZD1222 (Figure 3B) shows slightly different behaviour, since IgGs, with a few exceptions, raise above the threshold around day 21 and reach a maximum peak at day 28, with an average of 2.53 ± 2.38. Moreover, the boost with the second dose results in an important increase of IgG levels obtaining the maximum level at day 15 (with an average of 67.71 ± 22.45 for BNT162b2), while the highest level of IgGs for AZD1222 is reached at day 21 (with an average of 9.67 ± 8.36), as already reported in detail in Table S4. As expected, IgG levels start to decrease importantly four weeks after the boost for BNT162b2 and AZD1222 vaccines (of note, the last measured time point showed an IgG ratio of 25.03 ± 13.48 for BNT162b2 and of 7.07 ± 6.04 for AZD1222). It should be mentioned that the mean reported at each time point for the two groups were calculated excluding the 4 subjects (3 AZD1222 and 1 BNT162b2) that had been infected by SARS-CoV-2 months before vaccination. The RNI subjects are included in the bean plot of Figure 3 panels A and B, and as expected, some of them already have anti-SARS IgGs at time 0 and show an important increase in IgG levels already at day 7. In particular, day 7 after vaccination may be considered a discrimination point between people who have a previous natural infection and those who have never been infected. For both studied vaccines a decrease in the mean IgG ratio was observed after 42 days after the administration of the first dose.

Using a questionnaire, we also recorded the frequency of local as well as systemic side effects after the first dose of vaccines both for BNT162b2 and AZD1222 vaccinations (Figure S3). None of the studied participants reported severe vaccine reactions requiring hospitalization. However, the number of systemic side effects in AZD1222 treatment appear to be higher than the BNT162b2 ones, if comparing the first administrations. However, no correlation was found between the IgG ratio levels and the side effects reported (data not shown).

Regardless of the amount of produced antibodies, the percentage of subjects whose levels raise above the negative cut-off (cut-off = 1.2) at the various time points and for BNT162b2 and AZD1222 vaccines was evaluated and reported in Figure 3C,D, respectively, after the exclusion of the 4 seropositive patients above described. In particular, 43% of participants became positive ten days after BNT162b2 administration and 97% after 15 days (blue histograms in Figure 3C). Such a percentage is maintained at T21, before the second dose administration, and reaches 100% one week after the boost administration (II dose T7). On the other hand, only 4% of subjects receiving AZD1222 resulted positive ten days after the first dose, reaching 70.2% at day 35 (red histograms in Figure 3D). Intriguingly, the percentage of positive subjects decreases to 40% after 84 days of administration of AZD1222, just before the programmed second dose for this vaccine. It is interesting to note that, approximately 17 weeks after the first dose of vaccine, BNT162b2 and AZD1222, and after both second doses, a positive percentage of 100% was reached, as shown in the histograms in Figure 3C,D (of note some cases are just above the cut-off highlighting a little variation of the positive percentage in the following weeks).

**Figure 4:**
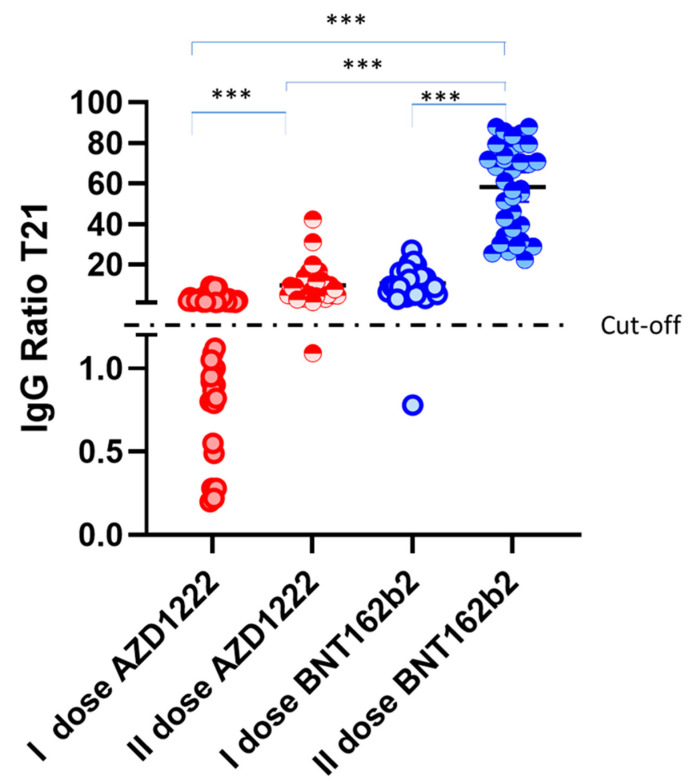
IgG ratio at 21 days of vaccines administration for both vaccines before and after the second dose administration. Kruskal–Wallis tests shows *p* value < 0.0001 (indicated with ***) for each comparison with the exception of II dose AZD1222 vs. first dose of BNT162b2. The figure shows as the second dose of AZD1222 results in an IgG ratio similar to the first dose of BNT162b2 (no significance of Kruskal–Wallis test).

Figure 4 focuses on the 21st day after the first and the second dose for both the vaccines, highlighting as the II AZD1222 dose IgG ratio overlaps with the I BNT162b2 dose. Of note, the only AZD1222 vaccinated donors resulting seronegative at day 21 after the second dose, was slightly positive at day 15, demonstrating a transient but effective vaccine response.

### 3.4. SARS-CoV-2–Specific T Cells in Vaccinated Subjects

**Figure 5:**
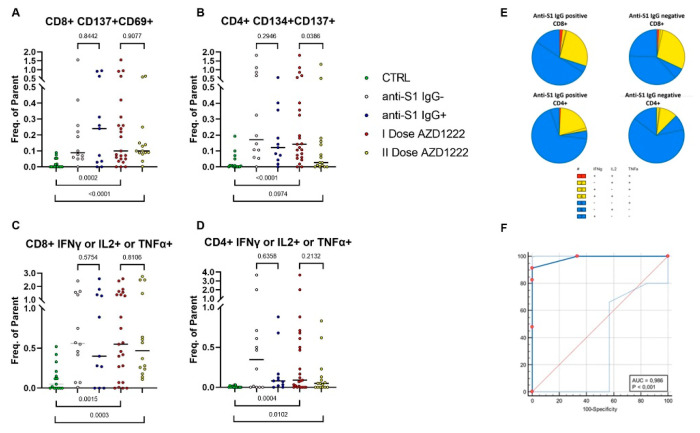
SARS-CoV-2-specific T cells in anti-S1 IgG seronegative and seropositive AZD1222 vaccinated donors. Frequencies of CD8+ or CD4+ T cells expressing T-cell activation markers (**A**,**B**) and producing antigen-specific cytokines (**C**,**D**) are shown in SARS-CoV-2 unexposed healthy donors who never received any anti-SARS-CoV-2 vaccine (CTRL), in anti-S1 IgG seronegative (anti-S1 IgG-) and seropositive (anti-S1 IgG+) donors vaccinated with a single dose of AZD1222, in the whole cohort of donors after a single dose of the AZD1222 vaccine administration (I dose AZD1222) and in a group of AZD1222 fully vaccinated donors. Frequencies were obtained by subtracting to the values of the stimulated samples the background produced by the related unstimulated tube. Individual data points are represented as scatter dot plots with lines showing the median value. Significance was calculated using non-parametric Mann–Whitney U-tests (two-tailed). (**E**) Pie charts show the expression of cytokine combinations from CD4+ and CD8+ T cells anti-S1 IgG seronegative and seropositive donors vaccinated with a single dose of AZD1222. (**F**) The ROC curve represents the performance of flow cytometry analysis.

Of 52 AZD1222 vaccinated subjects, analysed weekly for 17 weeks, 12 were anti-S1 IgG negative (anti-S1 IgG-) at any time point between the first and the second dose. In these subjects, we studied the adaptive immune responses using the TCR-dependent activation-induced marker (AIM) assay to quantify SARS-CoV-2-specific CD4+ and CD8+ T cells. The frequencies of CD134+CD137+ and CD69+CD137+ after the specific stimulation with the mix of S peptides were evaluated in this group and compared with a group of 11 seropositive AZD1222 vaccinated donors (anti-S1 IgG+). Of note, both groups displayed spike-protein reactive T cells (Table S5, Figure 5A,B). Flow cytometry with intracellular cytokine staining assays (ICS) of PBMCs from vaccinated donors, after a single dose of AZD1222, stimulated with the S peptide pool, also demonstrated antigen-specific cytokine secretion from both CD4+ and CD8+ T-cell compartments (Figure 5C,D, Tables S5 and S6). No statistical differences in terms of SARS-CoV-2–specific T cell frequencies were detected between the two groups both in terms of T cell memory markers (Figure 5A,B, Tables S5 and S6) and cytokine production (Figure 5C,D, Tables S5 and S6). In both groups only a few multifunctional CD4+ or CD8+ T cells were detected by the cytokine combination analysis, suggesting the development of a functional response (Figure 5E). Indeed, responses were dominated by T cells expressing single cytokines, as already reported [12]. As shown in Figure 5A–D, the whole cohort of analysed AZD1222 vaccinated donors (N = 23, I Dose AZD1222) was also compared to a group of SARS-CoV-2-unexposed healthy donors (negative controls, N = 15, CTRL), who never received any anti-SARS-CoV-2 vaccine and to a group of AZD1222 fully vaccinated donors (positive controls, N = 15, II dose AZD1222). T cell memory markers and cytokine production were significantly higher in donors vaccinated with a single dose of AZD1222 than in control subjects (Figure 5A,B, Tables S5 and S6). These data also demonstrated that the CD8 response tends to remain stable after the second dose of the vaccine, while the frequencies of CD4+ T cells expressing memory markers are more affected by the inter-subject variability. The ROC analysis was carried out comparing six SARS-CoV-2-unexposed healthy donors who never received any anti-SARS-CoV-2 vaccine and 23 volunteers after they had received the first dose of the AZD1222 vaccine (Figure 5F). This analysis, based on a flow cytometry binary score, generated by the single analysed parameters (CD4+ and CD8+ AIM and cytokine production) (Figure 5F) allows defining a related cut-off (>1) with high sensitivity (91.30%) and specificity (100%).

The only BNT162b2 subject resulting seronegative after the first vaccine dose also displayed SARS-CoV-2-specific CD4+ and CD8+ T cells (Figure S1D,E).

## 4. Discussion

Following the implementation of vaccination programs, effective sero-surveillance is in great demand. In this study, we used a fully automated high-throughput DELFIA immunoassay on DBS samples collected from a finger prick to evaluate anti-SARS-CoV-2 IgGs both in a large cohort of subjects as well as for a time-course study in a smaller cohort of subjects vaccinated with either AZD1222 or BNT162b2. We show that this is a reliable assay, an ideal tool for large-scale monitoring of the immune response, even among children, the elderly and frail patients [4,5]. Indeed, sample collection is simple allowing self-sampling, samples are stable for several weeks at room temperature [4] and can be shipped to the laboratory also from distant collection locations. Finally, analysis is high-throughput (up to 3600 samples per day can be analysed) and less expensive than many other methods for antibody detection.

Our time-course study shows that there is different behaviour in terms of IgG production between the two vaccines. The BNT162b2 vaccine results in higher IgG levels (10.79 ± 5.9) as compared to AZD1222 (2.10 ± 2.16) measured at 21 days after the first dose, showing a significantly different IgG level (Figure 4). As expected, the boost with the second dose results in much higher levels of IgGs as well as a more homogenous response as shown by the suppression of the standard deviation. Our data also suggest that the evaluation of IgG levels should be conducted at 21 days after the first dose and at 15 days after the second dose for the BNT162b2 vaccine, while for the AZD1222 vaccine the maximum IgG ratio is reached between day 28 and 35 after the first dose, and at 15 days after the second dose.

Between the first and the second dose, 30% of AZD1222 vaccinated subjects and 2.7% of BNT162b2 vaccinated subjects were negative at any tested time point. Our results, however, show that these subjects display a broad T cell response to the S protein, dominated by the secretion of single cytokines. Being generally accepted that the presence of cytotoxic CD8+ T cell responses, together with Th1-biased CD4+ effector responses give immune protection against SARS-CoV-2 exposure, these data strongly suggest the efficacy of the BNT162b2 and AZD1222 vaccines already after the first dose. Therefore, after the boost, 100% of the vaccinated donors resulted to have a positive IgG ratio. Of note, only one subject, AZD1222 vaccinated donor, showed an IgG ratio very close to the positive cut-off, resulting as negative at 21 days after the second dose (Figure 4). This subject showed a broad T cell response to the S protein. Moreover, the T cell response is known to last for at least six years [10] suggesting that the vaccination campaign will result in long-lasting protection. To this end, it will be interesting to continue monitoring both antibody levels as well as T cell response at later time points to confirm durable protection.

## 5. Conclusions

Our data show that despite the variability in levels of IgG produced and the time required for production, both vaccines are capable of generating an immune response already after the first dose and are potentially protective against severe disease. Of course, such an immune response is reinforced after the boost, showing 100% of positivity 15 days after the second dose. For the first time, our study details the antibody fluctuation after the two vaccines are administrated, and thus suggests the exact time-point for the evaluation of the antibody response to vaccination.

## Data Availability

Data is contained within the article and Supplementary Materials.

## Supplementary Materials

The following are available online at https://www.mdpi.com/article/10.3390/biomedicines9081035/s1, Figure S1: Gating strategy for SARS-CoV-2 S-reactive T cell identification after the first dose of vaccines, Figure S2: Dot plot of the four groups analysed, Figure S3: Side effects of SARS-CoV-2 Vaccines, Table S1: Summary of subjects enrolled in the study groups for each analysis, Table S2: Reagent List for Flow Cytometry Analyses, Table S3: BNT162b2 statistical results, Table S4: AZD1222 statistical results, Table S5: CD4+ Spike-reactive T-cells in seronegative and seropositive AZD1222 vaccinated donors, Table S6: CD8+ Spike-reactive T-cells in control and AZD1222 vaccinated donors.

## References

[1] Polack F.P., Thomas S.J., Kitchin N., Absalon J., Gurtman A., Lockhart S., Perez J.L., Perez Marc G., Moreira E.D., Zerbini C. (2020). Safety and Efficacy of the BNT162b2 mRNA COVID-19 Vaccine. N. Engl. J. Med..

[2] Voysey M., Clemens S.A.C., Madhi S.A., Weckx L.Y., Folegatti P.M., Aley P.K., Angus B., Baillie V.L., Barnabas S.L., Bhorat Q.E. (2021). Safety and efficacy of the ChAdOx1 nCoV-19 vaccine (AZD1222) against SARS-CoV-2: An interim analysis of four randomised controlled trials in Brazil, South Africa, and the UK. Lancet.

[3] Knoll M.D., Wonodi C. (2021). Oxford-AstraZeneca COVID-19 vaccine efficacy. Lancet.

[4] Brown L., Byrne R.L., Fraser A., Owen S.I., Cubas-Atienzar A.I., Williams C.T., Kay G.A., Cuevas L.E., Fitchett J.R.A., Fletcher T. (2021). Self-sampling of capillary blood for SARS-CoV-2 serology. Sci. Rep..

[5] Schultz J.S., McCarthy M.K., Rester C., Sabourin K.R., Annen K., DomBourian M., Eisenmesser E., Frazer-Abel A., Knight V., Jaenisch T. (2021). Development and validation of a multiplex microsphere immunoassay using dried blood spots for SARS-CoV-2 seroprevalence: Application in first responders in Colorado, USA. J. Clin. Microbiol..

[6] Gobbi F., Buonfrate D., Moro L., Rodari P., Piubelli C., Caldrer S., Riccetti S., Sinigaglia A., Barzon L. (2021). Antibody Response to the BNT162b2 mRNA COVID-19 Vaccine in Subjects with Prior SARS-CoV-2 Infection. Viruses.

[7] Amendola A., Bianchi S., Gori M., Barcellini L., Colzani D., Canuti M., Giacomet V., Fabiano V., Folgori L., Zuccotti G.V. (2021). Dried Blood Spot as an Alternative to Plasma/Serum for SARS-CoV-2 IgG Detection, an Opportunity to Be Sized to Facilitate COVID-19 Surveillance Among Schoolchildren. Pediatr. Infect. Dis. J..

[8] Zava T.T., Zava D.T. (2021). Validation of dried blood spot sample modifications to two commercially available COVID-19 IgG antibody immunoassays. Bioanalysis.

[9] Braun J., Loyal L., Frentsch M., Wendisch D., Georg P., Kurth F., Hippenstiel S., Dingeldey M., Kruse B., Fauchere F. (2020). SARS-CoV-2-reactive T cells in healthy donors and patients with COVID-19. Nature.

[10] Sauer K., Harris T. (2020). An Effective COVID-19 Vaccine Needs to Engage T Cells. Front. Immunol..

[11] Aiello A., Najafi Fard S., Petruccioli E., Petrone L., Vanini V., Farroni C., Cuzzi G., Navarra A., Gualano G., Mosti S. (2021). Spike is the most recognized antigen in the whole-blood platform in both acute and convalescent COVID-19 patients. Int. J. Infect. Dis. IJID Off. Publ. Int. Soc. Infect. Dis..

[12] Ewer K.J., Barrett J.R., Belij-Rammerstorfer S., Sharpe H., Makinson R., Morter R., Flaxman A., Wright D., Bellamy D., Bittaye M. (2021). T cell and antibody responses induced by a single dose of ChAdOx1 nCoV-19 (AZD1222) vaccine in a phase 1/2 clinical trial. Nat. Med..

[13] Tarke A., Sidney J., Kidd C.K., Dan J.M., Ramirez S.I., Yu E.D., Mateus J., da Silva Antunes R., Moore E., Rubiro P. (2020). Comprehensive analysis of T cell immunodominance and immunoprevalence of SARS-CoV-2 epitopes in COVID-19 cases. Biorxiv Prepr. Serv. Biol..

