# Supplementary material for: Picture of the Favourable Immune Profile Induced by Anti-SARS-CoV-2 Vaccination
Source: Biomedicines. 2021 Aug 18;9(8):1035. doi: 10.3390/biomedicines9081035 (PMC8391252; doi:10.3390/biomedicines9081035)
Supplement: Supplementary file 1 [file biomedicines-09-01035-s001.zip › biomedicines-1335467-supplementary.pdf]

biomedicines

MDPI

Methods

Patients

1269 subjects were enrolled, as reported in Table S1. Among these donors, 1181 were evaluated at a single time point, and were divided in four groups (2-70 years old); more specifically 398 individuals received one dose and 149 received two doses of AZD1222 vaccine, 257 received two doses of c vaccine, 235 subjects had a resolved natural SARS-CoV-2 infection, and 147 donors were non vaccinated and did not report previous infection (control group).

Moreover, an independent casuistry of 88 donors was weekly monitored for 77 days. These 88 volunteers underwent a time course study and IgG levels were evaluated before and after the administration of the vaccines. In detail, 36 participants (75% female, mean age 35,1±9.4 [range 23 to 61]), who received the first and second BNT162b2 vaccine doses, were monitored; among them only one donor was seropositive for COVID-19. Furthermore, 52 participants (59.6% female, mean age 40.8±9.2 [range 25 to 59]) receiving the first AZD1222 dose were also observed; among them three donors were seropositive for COVID-19. For the flow cytometry detection of spike-specific T cells, a total of 54 analyses was carried out. In detail, as specified in Table S1, 15 volunteers who were non vaccinated and did not report any previous SARS-CoV-2 infection (control group) were paralleled to a cohort of BNT162b2 and AZD1222 vaccinated individuals.

Table S1. Summary of subjects enrolled in the study groups for each analysis.

Study Design						
**Entry**	**Single Time Points**	**Age (Years)**	**Multiple Time Points**	**Age (Years)**	**T Memory Cells**	**Age (Years)**
AZD1222	547	48.86 ± 9.63	52	40.8 ± 9.2	38	43.34 ± 7.90
BNT162b2	252 (only 4 patients with a single dose)	48.15 ± 10.63	36	35.1 ± 9.4	1	61
RNI (Resolved Natural Infection)	235	45.97 ± 21.27	NA	NA	NA	NA
Negative Control	147	48.24 ± 16.22	NA	NA	15	45.93 ± 11.84
TOTAL	1181		88		54	

NA = not applicable

Functional Data Analysis, FDA

The basic idea of Functional Data Analysis (FDA) is to consider one or more observations as curves or functions, instead of scalar data. Therefore, we analysed the behaviour of IgG as a grow curve observed for each unit representing the individuals treated with a specific vaccine, more precisely BNT162b2 and AZD1222 vaccines. We also consider that the units represent a random sample of the observed curves.

The dataset used to implement the FDA approach is a data-matrix of the form:

$$x_n(t_{j,n}) \in \mathbb{R},\ \ t_{j,n} \in [T_1, T_2],\ \ n = 1, 2, \dots, N \quad j = 1 \dots, J_n \quad (1)$$

where $[T_1, T_2]$ define the time interval of observation, $n = 1, 2, \dots, N$ represents patients receiving the vaccine, and j denotes the specific time points whose each IgG is observed.

In our analysis, the smooth curves are defined as follow:

$$IgG_n(t), \quad t \in [T_1, T_2], \quad n = 1, 2, \dots N \qquad (2)$$

In particular, T_1 is the starting point of the observations, T_2 is the last point of observations; $N = N_1 + N_2$ are the sample units observed, where N_1 =37 refers to the individuals treated with Pfizer vaccine and N_2=50 indicates the individuals treated with AZD1222 injection.

The concentration of $IgG_n(t)$ occurs at any point t, but is measured exclusively at selected time t, corresponding to 0 (T0= 0), 7 (T7= 7), 14 (T14= 14) and 21 (T21= 21) days after the injection of the vaccine.

From an analytical point of view, the functional data reconstruction, defined in Equation 2, is obtained by using a basis expansion, as follow:

$$IgG_n(t) \approx \sum_{m=1}^{M} c_{nm} B_m(t), \quad 1 \le n \le N \qquad (3)$$

where c_{nm} represent the coefficient and B_m defines a standard basis functions, such splines, wavelets, or sine and cosine functions.

In our setting, we use B-splines basis, a polynomial approximation between couple of knots at each time of coordinates $[x_n(t_{j,n});\ t_{j,n}],\ \forall\, t_{j,n}$.

In our FDA of vaccine data, we first construct functional boxplots to displaying the main descriptive statistics. As known, the statistics used are robust and not affected by outliers.

Like to the classic boxplot for scalar data, the box represents the 50% of the observations and is delimited by the first and third quartiles. The line dividing the box is the median. The height of this area is called the interquartile range that can be viewed as an approximation of variability of the curves.

The functional boxplots are very informative exploratory tools for functional data, as they simultaneously allow evaluating the behaviour over time of all the statistics.

In the second step of our study, we go through the inferential analysis.

To this end, we need to assume that the function $IgG_n(t)$ are elements of a functional stochastic process in the Hilbert space L^2. In order to ensure that this condition is true, the observed data have to be transformed in functional objects deriving from Equation 3.

In this setting, each function can be view as a realization of a stochastic process in a Hilbert space L^2, with mean function:

$$\mu(t) = E(IgG(t)) \quad (4)$$

and covariance function:

$$G(t,s) = cov[IgG(t), IgG(s)] = E[(IgG(t) - \mu(t))(IgG(s) - \mu(s))] \quad (5)$$

The counterpart estimates of (4) and (5) are obtained as follow:

$$\overline{IgG}(t) = \frac{1}{n}\sum_{i=1}^{n} IgG\,(t) \quad (6)$$

$$\widetilde{G}(t,s) = \frac{1}{n-1}\sum_{i=1}^{n}(IgG(t) - \overline{IgG}(t))(IgG(s) - \overline{IgG}(s)) \quad (7)$$

Under the above hypothesis that function $IgG_n(t)$ are elements of a functional stochastic process in the Hilbert space L^2, Cao et al. [3], Ma et al. [4], Wang and Yang [5] proved that, for $n \to \infty$, the mean function estimator converges to $\sqrt{n}\,(\overline{IgG}(t) - \mu(t))$ in L^2 to a mean zero Gaussian process $W(t)$, for $t \in T$, with covariance function $E[W(t)W(s)] = G(t,s) = \{G(t,s)G(t,s)\}^{-1/2}$. Thus, the construction of the simultaneous confidence bands ([2]) is based on the supremum distribution of the Gaussian process, $sup_t W(t)$. In particular, as $n \to \infty$, $\forall\, \alpha \in (0,1)$, the mean curve estimator $\overline{IgG}(t)$, converges at the $\sqrt{n}$ rate:

$$P\left\{sup_{t\in\chi} n^{\frac{1}{2}} |\overline{IgG}(t) - \mu(t)| \widetilde{G}(t,s)^{-\frac{1}{2}} \le Q_{1-\alpha}\right\} \to 1 - \alpha \quad (8)$$

$$P\left\{n^{\frac{1}{2}}|\overline{IgG}(t)-\mu(t)|\widetilde{G}(t,s)^{-\frac{1}{2}} \le Z_{1-\alpha/2}\right\} \to 1-\alpha, \forall t \in \chi \quad (9)$$

where $Q_{1-\alpha}$ is the 100 (1-α)th percentile of the absolute maxima distribution of $W(t)$ and Z $_{1-\alpha/2}$ is the 100 (1-α/2)th percentile of the standard normal distribution. Therefore, as $n \to \infty$, an asymptotic 100 (1-α) % correct confidence band for $\mu(t)$ is:

$$\overline{IgG}(t) \pm \widetilde{G}(t,s)^{1/2} Q_{1-\alpha} n^{-1/2} \quad (10)$$

While an asymptotic 100 (1-α) % pointwise confidence interval for $\mu(t)$ is:

$$\overline{IgG}(t) \pm \widetilde{G}(t,s)^{1/2} Z_{1-\alpha/2} n^{-1/2} \quad (11)$$

The generation of neutralizing antibodies cooperates with B- and T-cell responses in the adaptive immune responses directed against the spike glycoprotein (S)[8], inducing long-term protection from severe respiratory infection (> 6-17 years) [9]. This long-lasting antiviral immunity requires the enrolment of T-cells, both CD4+ and CD8+, and the generation of effective T cell memory that can also serve as a sensitive biomarker of previous exposures to the spike glycoprotein [9]. We therefore also evaluated T-cell response in subjects seronegative after vaccine administration.

PBMC isolation, Stimulation and Staining for Flow Cytometry Analysis

Peripheral blood mononuclear cells (PBMC) were obtained from 8 ml of peripheral blood, using sodium citrate cell preparation tube (BD Biosciences, San Jose, CA). PBMC were harvested and seeded in RPMI-1640 medium (containing 10% Foetal Bovine Serum, 1% Penicillin-Streptomycin and 10 mM L-Glutamine) at the concentration of 2x10^6 cells/ml. 2x10^6 cells were stimulated with a pool of spike peptides (PepTivator S, cat. 130-126-701, PepTivator S1, cat. 130-127-048, Peptivator S+, cat. 130-127-312, Miltenyi Biotec, Bergisch Gladbach, Germany) at the recommended concentrations, for 16 h (37°C, 5 % of CO2), while negative controls were treated with the same amount of vehicle *[PMID: 33864921], [PMID: 32726801; PMID: 33335323].* After 2 h of stimulation, samples were treated with 6.5 µl GolgiStop (554724, BD Biosciences). PBMCs were incubated for 30 minutes at room temperature in the dark with the surface antibodie mix, as reported in Table S2. Cells were then fixed and permeabilized by adding 250 µl of Cytofix/Cytoperm solution (BD Biosciences), and then stained for 30 minutes with the intracellular mix of antibodies (Table S2). Samples were finally washed and 3.5 x 10^5 cells/sample were acquired by flow cytometry (CytoFLEX, Beckman Coulter, Brea, CA). TCR-dependent activation induced marker (AIM) assay *[PMID: 33330869]* and flow cytometry with intracellular cytokine staining assays (ICS) were carried out and analysed as reported *[PMID: 33330869; PMID: 33335323].* The background was removed from the data by subtracting the single percentage of AIM+ or cytokine+ cell frequencies of the sample stimulated with DMSO (vehicle only). For each donor, the gates for AIM+ and cytokine+ cells were drawn based on the related negative control. A representative example of the used gating strategy is depicted in Figure S1.

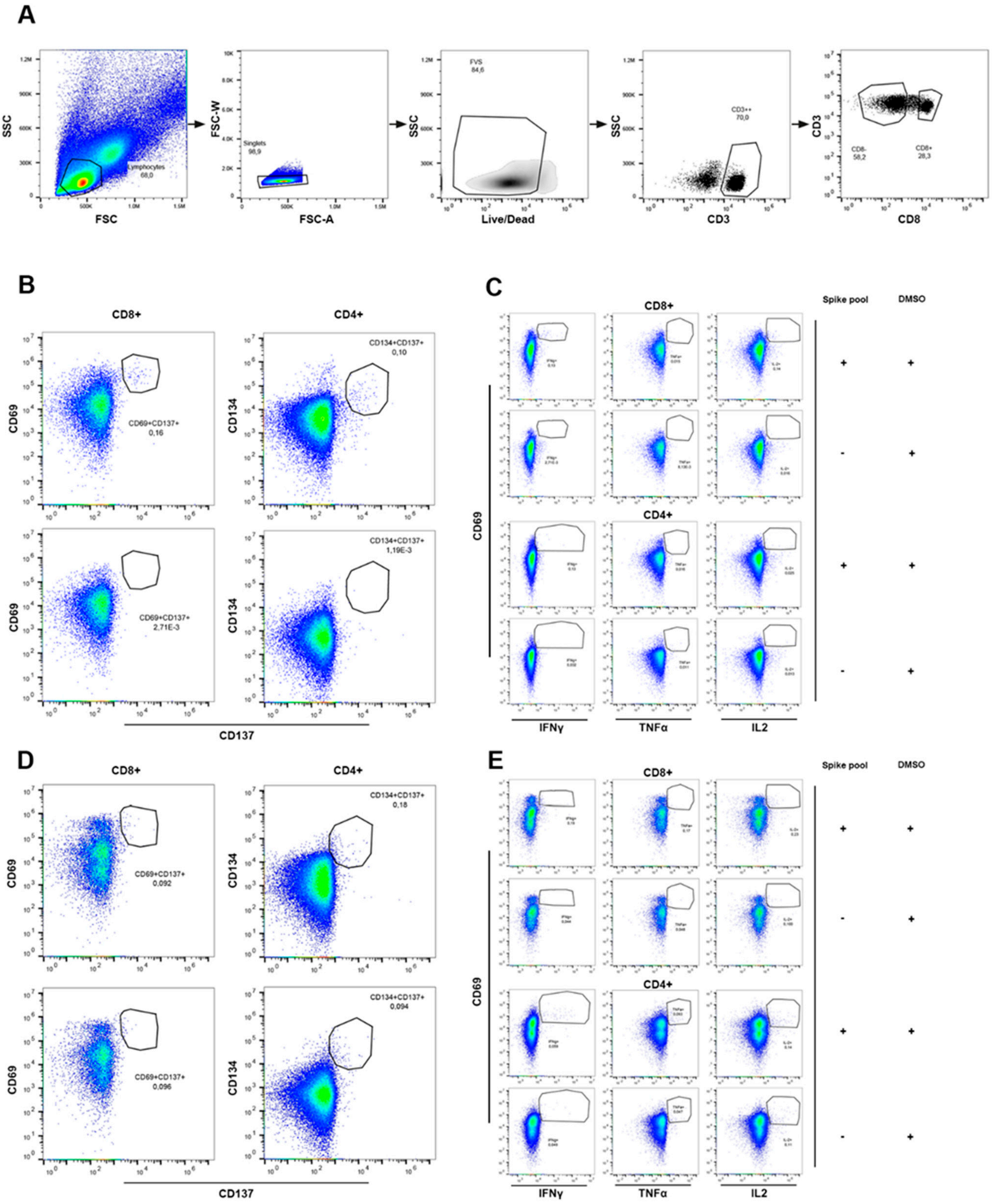

Figure S1. Gating strategy for SARS-CoV-2 S-reactive T cell identification after the first dose of vaccines. Gating strategy to detect SARS-CoV-2 S-reactive CD8+ and CD4+ T cells after their in vitro stimulation with SARS-CoV-2 S peptide pools for 16 h. Representative images of one donor after the first dose of vaccine are shown. (**A**) Lymphocytes were first selected in a forward scatter (FSC) area versus side scatter (SSCA) area gate. Next, aggregates were excluded (FSC-A/FSC-width [W] plot) and live cells were identified (FVS- events). CD3+ T-cells were then gated (CD3/SSCA dot-plot). T-cells were split into CD8- (further identified as CD4+ cells) and CD8+ subsets (CD8/CD3). (**B**) TCR-dependent activation induced

markers (AIM) from a representative donor after the first dose of AZD1222 vaccine are represented, using the corresponding dimethylsulfoxide (DMSO) control to assess and subtract the background. (**C**) Cytokines (interferon [IFN]-γ, CD40L, tumor necrosis factor [TNF]-α and interleukin [IL]-2) were individually analyzed for each subset (CD4+ and CD8+), in a representative AZD1222 vaccinated donor, using the corresponding DMSO control to assess and subtract the background. (**D**) AIM+ CD4+ T and CD8+ T cell reactivity in BNT162b2 cases between the negative control (DMSO) and antigen-specific stimulations. (**E**) Antigen-specific cytokine production was detected in BNT162b2 donors after stimulation with the spike peptide pool or the negative control (DMSO).

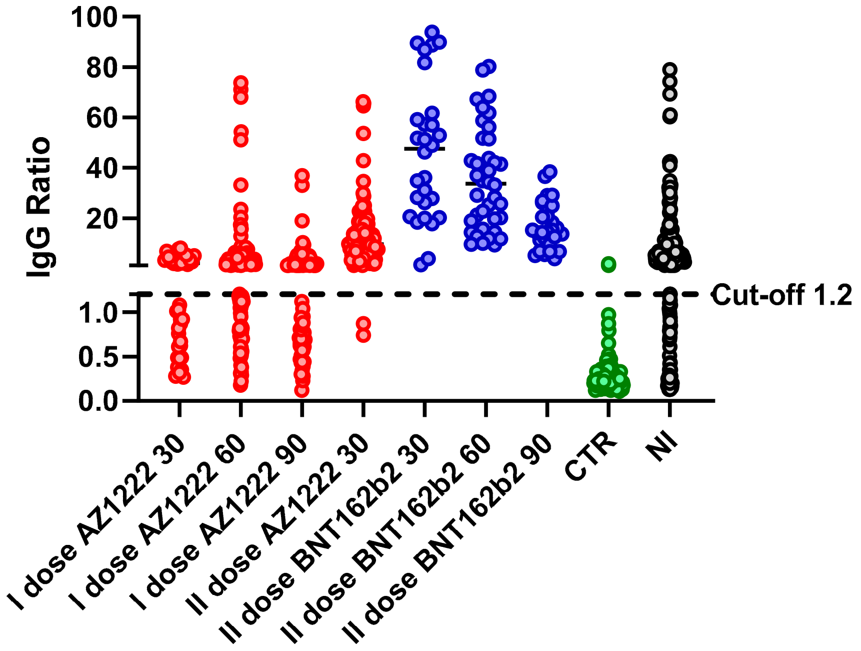

Figure S2. Dot plot of the four groups analysed: Red dots IgG ratio for AZD1222 vaccinated donors, blue dots for BNT162b2 vaccinated donors, white RNI donors and green dots controls subjects, divided into subgroup of 30 days depending on the time elapsed from the start of vaccination. 30 days, 60 days and 90 days were reported.

Table S2. Reagent List for Flow Citometry Analyses.

Specifity	Clone	Fluorochrome	Amount *per* test	Type staining	Catalogue Number
CD197	2-L1-A	PE	5 µl	Surface	566742
CD3	SK7	PerCP-Cy5.5	20 µl	Surface	332771
CD69	FN50	PE-Cy7	5 µl	Surface	561928
CD45RA	HI100	APC-H7	5 µl	Surface	560674
CD8	SK1	V500-C	5 µl	Surface	647457
FVS 575V	NA	NA	1 µl	Surface	565694
CD134	L106	BV768	5 µl	Intracellular	744746
CD137	4B4-1	APC	20 µl	Intracellular	550890
IFNg	B27	FITC	20 µl	Intracellular	552887
TNFa	MAb11	Alexa Fluor™700	5 µl	Intracellular	557996
IL-2	5344.111	BV711	5 µl	Intracellular	563946

R-phycoerythrin (PE); peridinin chlorophyll protein-cyanine 5.5 (PerCP-Cy 5.5); PE-Cyanine 7 (Cy7); Allophycocyanin-Hilite®7 (APC-H7); Fixable Viability Stain (FVS); Brilliant Violet (BV); Allophycocyanin (APC); Fluorescein isothiocyanate (FITC). All the reagents listed in the table are from BD Bdiosciences.

NA = not applicable

Flow Cytometry analyses

Instrument performances and data reproducibility were sustained and checked by using the beads CytoFLEX Daily QC Fluorospheres (ref. B53230, Beckman Coulter). To assess non-specific fluorescence, fluorescence minus one (FMO) controls were used. Compensation was calculated using VersaComp Antibody Capture Beads (ref B22804, Beckman Coulter) and single stained samples. Data were analyzed using FlowJo v 10.7.2 (BD Biosciences) and SPICE v 6.1 (provided by M. Roederer, National Institutes of Health; Roederer) software. Background subtraction was performed using PESTLE v 2.0 software (provided by M. Roederer, National Institutes of Health). Functional subsets were obtained by boolean gating. Frequencies of T-cell responses were displayed as percentage of CD4+ or CD8+ T-cells. T-cells producing at least 1 of the tested cytokines in the CD4+ and CD8+ T-cell compartments were considered specific for S protein stimulation.

IgG level Statistics

Kruskal-Wallis test was applied to multiple time points of IgG measurement, than a Dunn's multiple comparison test was applied as post-hoc test, details are reported in Table S3 for BNT162b2 and Table S4 for AZD1222. Not significant test is reported as "ns", while p<0.05 is reported as *, p<0.01 is reported as ** and p<0.001 is reported as ***. Seropositive patients were excluded from the statistical analyses.

Table S3. BNT162b2 statistical results.

Dunn's Multiple Comparisons Test	Mean Rank Diff	Significance	Summary	Adjusted P Value
T0 vs. T7	-24,48	No	ns	>0,9999
T0 vs. T10	-64,97	No	ns	>0,9999
T0 vs. T15	-131,2	Yes	**	0,0073
T0 vs. T21	-156,7	Yes	***	0,0002
T0 vs. II DOSE T7	-373,3	Yes	****	<0,0001
T0 vs. II DOSE T14	-395,9	Yes	****	<0,0001
T0 vs. II DOSE T21	-368,7	Yes	****	<0,0001
T0 vs. II DOSE T28	-356,9	Yes	****	<0,0001
T0 vs. II DOSE T35	-315,7	Yes	****	<0,0001
T0 vs. II DOSE T49	-268,3	Yes	****	<0,0001
T0 vs. II DOSE T63	-243,4	Yes	****	<0,0001
T0 vs. II DOSE T77	-207,1	Yes	****	<0,0001
T0 vs. II DOSE T91	-202,8	Yes	****	<0,0001
T7 vs. T10	-40,5	No	ns	>0,9999
T7 vs. T15	-106,7	No	ns	0,0964
T7 vs. T21	-132,3	Yes	**	0,0045
T7 vs. II DOSE T7	-348,8	Yes	****	<0,0001
T7 vs. II DOSE T14	-371,4	Yes	****	<0,0001
T7 vs. II DOSE T21	-344,2	Yes	****	<0,0001
T7 vs. II DOSE T28	-332,4	Yes	****	<0,0001
T7 vs. II DOSE T35	-291,3	Yes	****	<0,0001
T7 vs. II DOSE T49	-243,8	Yes	****	<0,0001
T7 vs. II DOSE T63	-218,9	Yes	****	<0,0001
T7 vs. II DOSE T77	-182,7	Yes	****	<0,0001
T7 vs. II DOSE T91	-178,4	Yes	***	0,0003
T10 vs. T15	-66,21	No	ns	>0,9999
T10 vs. T21	-91,77	No	ns	0,4964
T10 vs. II DOSE T7	-308,3	Yes	****	<0,0001
T10 vs. II DOSE T14	-330,9	Yes	****	<0,0001
T10 vs. II DOSE T21	-303,7	Yes	****	<0,0001
T10 vs. II DOSE T28	-291,9	Yes	****	<0,0001
T10 vs. II DOSE T35	-250,8	Yes	****	<0,0001
T10 vs. II DOSE T49	-203,3	Yes	****	<0,0001
T10 vs. II DOSE T63	-178,4	Yes	****	<0,0001
T10 vs. II DOSE T77	-142,2	Yes	**	0,0053
T10 vs. II DOSE T91	-137,9	Yes	*	0,034
T15 vs. T21	-25,56	No	ns	>0,9999
T15 vs. II DOSE T7	-242,1	Yes	****	<0,0001
T15 vs. II DOSE T14	-264,7	Yes	****	<0,0001
T15 vs. II DOSE T21	-237,5	Yes	****	<0,0001

T15 vs. II DOSE T28	-225,7	Yes	****	<0,0001
T15 vs. II DOSE T35	-184,6	Yes	****	<0,0001
T15 vs. II DOSE T49	-137,1	Yes	**	0,0039
T15 vs. II DOSE T63	-112,2	No	ns	0,074
T15 vs. II DOSE T77	-75,95	No	ns	>0,9999
T15 vs. II DOSE T91	-71,65	No	ns	>0,9999
T21 vs. II DOSE T7	-216,5	Yes	****	<0,0001
T21 vs. II DOSE T14	-239,1	Yes	****	<0,0001
T21 vs. II DOSE T21	-212	Yes	****	<0,0001
T21 vs. II DOSE T28	-200,1	Yes	****	<0,0001
T21 vs. II DOSE T35	-159	Yes	***	0,0001
T21 vs. II DOSE T49	-111,5	No	ns	0,0794
T21 vs. II DOSE T63	-86,63	No	ns	0,8851
T21 vs. II DOSE T77	-50,39	No	ns	>0,9999
T21 vs. II DOSE T91	-46,09	No	ns	>0,9999
II DOSE T7 vs. II DOSE T14	-22,6	No	ns	>0,9999
II DOSE T7 vs. II DOSE T21	4,571	No	ns	>0,9999
II DOSE T7 vs. II DOSE T28	16,41	No	ns	>0,9999
II DOSE T7 vs. II DOSE T35	57,53	No	ns	>0,9999
II DOSE T7 vs. II DOSE T49	105	No	ns	0,157
II DOSE T7 vs. II DOSE T63	129,9	Yes	**	0,0096
II DOSE T7 vs. II DOSE T77	166,1	Yes	***	0,0002
II DOSE T7 vs. II DOSE T91	170,4	Yes	***	0,0008
II DOSE T14 vs. II DOSE T21	27,17	No	ns	>0,9999
II DOSE T14 vs. II DOSE T28	39	No	ns	>0,9999
II DOSE T14 vs. II DOSE T35	80,13	No	ns	>0,9999
II DOSE T14 vs. II DOSE T49	127,6	Yes	*	0,014
II DOSE T14 vs. II DOSE T63	152,5	Yes	***	0,0006
II DOSE T14 vs. II DOSE T77	188,7	Yes	****	<0,0001
II DOSE T14 vs. II DOSE T91	193	Yes	****	<0,0001
II DOSE T21 vs. II DOSE T28	11,83	No	ns	>0,9999
II DOSE T21 vs. II DOSE T35	52,96	No	ns	>0,9999
II DOSE T21 vs. II DOSE T49	100,4	No	ns	0,3191
II DOSE T21 vs. II DOSE T63	125,3	Yes	*	0,0245
II DOSE T21 vs. II DOSE T77	161,6	Yes	***	0,0006
II DOSE T21 vs. II DOSE T91	165,9	Yes	**	0,0021
II DOSE T28 vs. II DOSE T35	41,12	No	ns	>0,9999
II DOSE T28 vs. II DOSE T49	88,59	No	ns	0,8216
II DOSE T28 vs. II DOSE T63	113,5	No	ns	0,0749
II DOSE T28 vs. II DOSE T77	149,7	Yes	**	0,0021
II DOSE T28 vs. II DOSE T91	154	Yes	**	0,0064
II DOSE T35 vs. II DOSE T49	47,47	No	ns	>0,9999
II DOSE T35 vs. II DOSE T63	72,37	No	ns	>0,9999
II DOSE T35 vs. II DOSE T77	108,6	No	ns	0,1934
II DOSE T35 vs. II DOSE T91	112,9	No	ns	0,3243
II DOSE T49 vs. II DOSE T63	24,9	No	ns	>0,9999
II DOSE T49 vs. II DOSE T77	61,14	No	ns	>0,9999
II DOSE T49 vs. II DOSE T91	65,44	No	ns	>0,9999
II DOSE T63 vs. II DOSE T77	36,24	No	ns	>0,9999

II DOSE T63 vs. II DOSE T91	40,54	No	ns	>0,9999
II DOSE T77 vs. II DOSE T91	4,298	No	ns	>0,9999

Table S4. AZD1222 statistical results. Seropositive patients were excluded from the statistical analyses.

Dunn's Multiple Comparisons Test	Mean Rank Diff	Significant	Summary	Adjusted P Value
T0 vs. T7	-1,533	No	ns	>0,9999
T0 vs. T10	-39,33	No	ns	>0,9999
T0 vs. T15	-176,3	Yes	*	0,0182
T0 vs. T21	-354,1	Yes	****	<0,0001
T0 vs. T28	-379,7	Yes	****	<0,0001
T0 vs. T35	-389,2	Yes	****	<0,0001
T0 vs. T42	-374,4	Yes	****	<0,0001
T0 vs. T49	-338,2	Yes	****	<0,0001
T0 vs. T56	-352,7	Yes	****	<0,0001
T0 vs. T63	-296,9	Yes	****	<0,0001
T0 vs. T70	-288,3	Yes	****	<0,0001
T0 vs. T77	-293,7	Yes	****	<0,0001
T0 vs. T84	-288,2	Yes	****	<0,0001
T0 vs. II DOSE T7	-530,2	Yes	****	<0,0001
T0 vs. II DOSE T15	-597,7	Yes	****	<0,0001
T0 vs. II DOSE T21	-587,4	Yes	****	<0,0001
T0 vs. II dose T28	-566,4	Yes	****	<0,0001
T7 vs. T10	-37,8	No	ns	>0,9999
T7 vs. T15	-174,7	Yes	*	0,0175
T7 vs. T21	-352,6	Yes	****	<0,0001
T7 vs. T28	-378,2	Yes	****	<0,0001
T7 vs. T35	-387,6	Yes	****	<0,0001
T7 vs. T42	-372,8	Yes	****	<0,0001
T7 vs. T49	-336,7	Yes	****	<0,0001
T7 vs. T56	-351,1	Yes	****	<0,0001
T7 vs. T63	-295,3	Yes	****	<0,0001
T7 vs. T70	-286,8	Yes	****	<0,0001
T7 vs. T77	-292,2	Yes	****	<0,0001
T7 vs. T84	-286,7	Yes	****	<0,0001
T7 vs. II DOSE T7	-528,6	Yes	****	<0,0001
T7 vs. II DOSE T15	-596,2	Yes	****	<0,0001
T7 vs. II DOSE T21	-585,9	Yes	****	<0,0001
T7 vs. II dose T28	-564,9	Yes	****	<0,0001
T10 vs. T15	-136,9	No	ns	0,3442
T10 vs. T21	-314,8	Yes	****	<0,0001
T10 vs. T28	-340,4	Yes	****	<0,0001
T10 vs. T35	-349,8	Yes	****	<0,0001
T10 vs. T42	-335,1	Yes	****	<0,0001
T10 vs. T49	-298,9	Yes	****	<0,0001
T10 vs. T56	-313,3	Yes	****	<0,0001
T10 vs. T63	-257,5	Yes	****	<0,0001
T10 vs. T70	-249	Yes	****	<0,0001

T10 vs. T77	-254,4	Yes	****	<0,0001
T10 vs. T84	-248,9	Yes	***	0,0001
T10 vs. II DOSE T7	-490,8	Yes	****	<0,0001
T10 vs. II DOSE T15	-558,4	Yes	****	<0,0001
T10 vs. II DOSE T21	-548,1	Yes	****	<0,0001
T10 vs. II dose T28	-527,1	Yes	****	<0,0001
T15 vs. T21	-177,8	Yes	*	0,0111
T15 vs. T28	-203,5	Yes	***	0,0009
T15 vs. T35	-212,9	Yes	***	0,0004
T15 vs. T42	-198,1	Yes	**	0,0021
T15 vs. T49	-161,9	No	ns	0,0577
T15 vs. T56	-176,4	Yes	*	0,0138
T15 vs. T63	-120,6	No	ns	>0,9999
T15 vs. T70	-112	No	ns	>0,9999
T15 vs. T77	-117,4	No	ns	>0,9999
T15 vs. T84	-112	No	ns	>0,9999
T15 vs. II DOSE T7	-353,9	Yes	****	<0,0001
T15 vs. II DOSE T15	-421,5	Yes	****	<0,0001
T15 vs. II DOSE T21	-411,2	Yes	****	<0,0001
T15 vs. II dose T28	-390,1	Yes	****	<0,0001
T21 vs. T28	-25,62	No	ns	>0,9999
T21 vs. T35	-35,07	No	ns	>0,9999
T21 vs. T42	-20,29	No	ns	>0,9999
T21 vs. T49	15,9	No	ns	>0,9999
T21 vs. T56	1,426	No	ns	>0,9999
T21 vs. T63	57,22	No	ns	>0,9999
T21 vs. T70	65,8	No	ns	>0,9999
T21 vs. T77	60,4	No	ns	>0,9999
T21 vs. T84	65,86	No	ns	>0,9999
T21 vs. II DOSE T7	-176,1	Yes	*	0,0369
T21 vs. II DOSE T15	-243,6	Yes	****	<0,0001
T21 vs. II DOSE T21	-233,3	Yes	***	0,0005
T21 vs. II dose T28	-212,3	Yes	**	0,01
T28 vs. T35	-9,452	No	ns	>0,9999
T28 vs. T42	5,336	No	ns	>0,9999
T28 vs. T49	41,52	No	ns	>0,9999
T28 vs. T56	27,05	No	ns	>0,9999
T28 vs. T63	82,84	No	ns	>0,9999
T28 vs. T70	91,42	No	ns	>0,9999
T28 vs. T77	86,03	No	ns	>0,9999
T28 vs. T84	91,48	No	ns	>0,9999
T28 vs. II DOSE T7	-150,4	No	ns	0,261
T28 vs. II DOSE T15	-218	Yes	***	0,0005
T28 vs. II DOSE T21	-207,7	Yes	**	0,0049
T28 vs. II dose T28	-186,7	No	ns	0,0684
T35 vs. T42	14,79	No	ns	>0,9999
T35 vs. T49	50,97	No	ns	>0,9999
T35 vs. T56	36,5	No	ns	>0,9999
T35 vs. T63	92,29	No	ns	>0,9999

T35 vs. T70	100,9	No	ns	>0,9999
T35 vs. T77	95,48	No	ns	>0,9999
T35 vs. T84	100,9	No	ns	>0,9999
T35 vs. II DOSE T7	-141	No	ns	0,5241
T35 vs. II DOSE T15	-208,6	Yes	**	0,0013
T35 vs. II DOSE T21	-198,3	Yes	*	0,0118
T35 vs. II dose T28	-177,2	No	ns	0,1374
T42 vs. T49	36,18	No	ns	>0,9999
T42 vs. T56	21,71	No	ns	>0,9999
T42 vs. T63	77,51	No	ns	>0,9999
T42 vs. T70	86,09	No	ns	>0,9999
T42 vs. T77	80,69	No	ns	>0,9999
T42 vs. T84	86,14	No	ns	>0,9999
T42 vs. II DOSE T7	-155,8	No	ns	0,208
T42 vs. II DOSE T15	-223,3	Yes	***	0,0004
T42 vs. II DOSE T21	-213,1	Yes	**	0,0039
T42 vs. II dose T28	-192	No	ns	0,0546
T49 vs. T56	-14,47	No	ns	>0,9999
T49 vs. T63	41,32	No	ns	>0,9999
T49 vs. T70	49,9	No	ns	>0,9999
T49 vs. T77	44,51	No	ns	>0,9999
T49 vs. T84	49,96	No	ns	>0,9999
T49 vs. II DOSE T7	-192	Yes	*	0,0121
T49 vs. II DOSE T15	-259,5	Yes	****	<0,0001
T49 vs. II DOSE T21	-249,2	Yes	***	0,0001
T49 vs. II dose T28	-228,2	Yes	**	0,0034
T56 vs. T63	55,79	No	ns	>0,9999
T56 vs. T70	64,37	No	ns	>0,9999
T56 vs. T77	58,98	No	ns	>0,9999
T56 vs. T84	64,43	No	ns	>0,9999
T56 vs. II DOSE T7	-177,5	Yes	*	0,0351
T56 vs. II DOSE T15	-245,1	Yes	****	<0,0001
T56 vs. II DOSE T21	-234,8	Yes	***	0,0004
T56 vs. II dose T28	-213,7	Yes	**	0,0095
T63 vs. T70	8,581	No	ns	>0,9999
T63 vs. T77	3,184	No	ns	>0,9999
T63 vs. T84	8,639	No	ns	>0,9999
T63 vs. II DOSE T7	-233,3	Yes	***	0,0007
T63 vs. II DOSE T15	-300,8	Yes	****	<0,0001
T63 vs. II DOSE T21	-290,6	Yes	****	<0,0001
T63 vs. II dose T28	-269,5	Yes	***	0,0002
T70 vs. T77	-5,397	No	ns	>0,9999
T70 vs. T84	0,05824	No	ns	>0,9999
T70 vs. II DOSE T7	-241,9	Yes	***	0,0001
T70 vs. II DOSE T15	-309,4	Yes	****	<0,0001
T70 vs. II DOSE T21	-299,1	Yes	****	<0,0001
T70 vs. II dose T28	-278,1	Yes	****	<0,0001
T77 vs. T84	5,455	No	ns	>0,9999
T77 vs. II DOSE T7	-236,5	Yes	***	0,0005

T77 vs. II DOSE T15	-304	Yes	****	<0,0001
T77 vs. II DOSE T21	-293,8	Yes	****	<0,0001
T77 vs. II dose T28	-272,7	Yes	***	0,0002
T84 vs. II DOSE T7	-241,9	Yes	***	0,0008
T84 vs. II DOSE T15	-309,5	Yes	****	<0,0001
T84 vs. II DOSE T21	-299,2	Yes	****	<0,0001
T84 vs. II dose T28	-278,1	Yes	***	0,0002
II DOSE T7 vs. II DOSE T15	-67,57	No	ns	>0,9999
II DOSE T7 vs. II DOSE T21	-57,29	No	ns	>0,9999
II DOSE T7 vs. II dose T28	-36,23	No	ns	>0,9999
II DOSE T15 vs. II DOSE T21	10,28	No	ns	>0,9999
II DOSE T15 vs. II dose T28	31,34	No	ns	>0,9999
II DOSE T21 vs. II dose T28	21,06	No	ns	>0,9999

Flow-cytometry statistics

Non-parametric Mann–Whitney U tests was used to compare cell frequencies between groups (two-tailed). P values less than 0.05 were considered significant. The ROC analysis was carried out calculating a flow cytometry binary score, obtained by the use of the criterion values and the coordinates of the single ROC curves generated by the single analyzed parameters (CD4+ and CD8+ AIM and cytokine production) between control and vaccinated subjects. The score was used to calculate the final ROC curve Using a questionnaire (https://docs.google.com/forms/d/e/1FAIpQLSd6oSebAuP56calMMcBOt-qYMK1AB4heF95TGQ8Y3b61KQgk2g/viewform), we also recorded the frequency of local as well as systemic side effects after the first dose of vaccines both for BNT162b2 and AZD1222 vaccinations (Figure S3). None of the studied participants reported severe vaccine reactions requiring hospitalization. However, the number of systemic side effects in AZD1222 treatment appear to be higher than the BNT162b2 ones, if comparing the first administrations. While the frequency of adverse symptoms after the second BNT162b2 dose is comparable to the one of AZD1222 first dose.

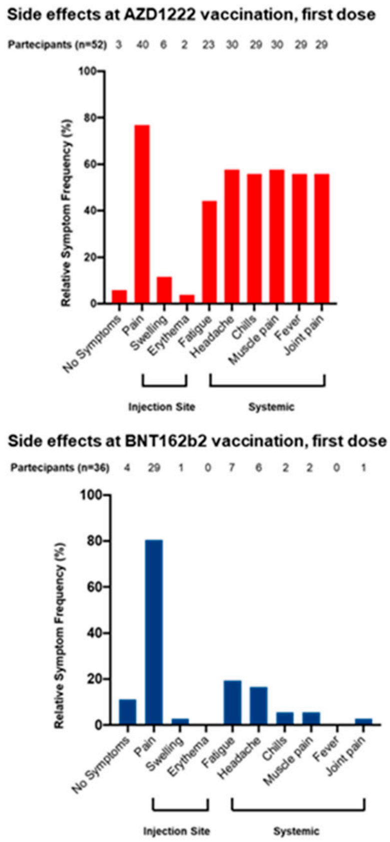

Figure S3. Side effects of SARS-CoV-2 Vaccines: Histograms represent the relative frequency of local as well as systemic vaccine-associated side effects. The bars show the relative frequency of each symptom (red for AZD1222 vaccination, first dose, blue for BNT162b2 vaccination, first dose), and the number reported at the top of the graphs indicate the absolute numbers for each symptom.

Table S5. CD4+ Spike-reactive T cells.

	CD4+ IFNg+ or IL2+ or TNFa+ CTRL	CD4+ IFNg+ or IL2+ or TNFa+ anti-S1 IgG positive	CD4+ IFNg+ or IL2+ or TNFa+ anti-S1 IgG negative	CD4+ IFNg+ or IL2+ or TNFa+ I Dose AZD1222	CD4+ IFNg+ or IL2+ or TNFa+ II Dose AZD1222	CD4+ CD134+ CD137+ anti-S1 IgG CTRL	CD4+ CD134+ CD137+ anti-S1 IgG positive	CD4+ CD134+ CD137+ anti-S1 IgG negative	CD4+ CD134+ CD137+ I Dose AZD1222	CD4+ CD134+ CD137+ II Dose AZD1222
**N**	15	11	12	23	15	15	11	12	23	15
**Minimum**	0.000	0.000	0.000	0.000	0.000	0.000	0.000	0.000	0.000	0.000
**Maximum**	0.030	0.880	3.670	3.670	0.830	0.192	0.570	1.820	1.820	1.310
**Median**	0.000	0.080	0.347	0.090	0.050	0.006	0.121	0.170	0.142	0.027
**Mean**	0.006	0.194	0.684	0.449	0.139	0.030	0.181	0.455	0.324	0.168
**SD**	0.011	0.297	1.099	0.840	0.249	0.054	0.183	0.561	0.438	0.343

Table S6. CD8+ Spike-reactive T cells.

	CD8+ IFNg+ or IL2+ or TNFa+ CTRL	CD8+ IFNg+ or IL2+ or TNFa+ anti-S1 IgG positive	CD8+ IFNg+ or IL2+ or TNFa+ anti-S1 IgG negative	CD8+ IFNg+ or IL2+ or TNFa+	CD8+ IFNg+ or IL2+ or TNFa+	CD8+ CD137+ CD69+ anti-S1 IgG CTRL	CD8+ CD137+ CD69+ anti-S1 IgG positive	CD8+ CD137+ CD69+ anti-S1 IgG negative	CD8+ CD137+ CD69+ I Dose AZD1222	CD8+ CD137+ CD69+ II Dose AZD1222

				I Dose AZD1222	II Dose AZD1222					
**N**	15	11	12	23	15	15	11	12	23	15
**Minimum**	0.000	0.000	0.010	0.000	0.110	0.000	0.000	0.000	0.000	0.003
**Maximum**	0.520	2.586	2.420	2.586	2.760	0.089	0.946	1.557	1.557	0.642
**Median**	0.050	0.400	0.560	0.550	0.470	0.000	0.240	0.088	0.100	0.100
**Mean**	0.129	0.808	0.825	0.817	0.902	0.020	0.312	0.254	0.282	0.177
**SD**	0.170	0.859	0.757	0.789	0.976	0.031	0.358	0.426	0.387	0.194
